# COVID 19 and the oxygen bottleneck

**DOI:** 10.2471/BLT.20.020920

**Published:** 2020-09-01

## Abstract

The COVID 19 pandemic is exposing an important weakness in health systems: medical oxygen production and delivery. Tatum Anderson reports.

Dr Tihitena (Tito) Negussie Mammo knows what it’s like to run out of oxygen. “Everything stops,” she says. “I have even had it happen in the middle of emergency surgery. When it does happen, we have to use a bag and valve mask, attaching a small portable oxygen cylinder if there’s one on hand.”

The most striking thing about Tito’s oxygen problem is the context in which it is occurring.

One of just 18 paediatric surgeons in Ethiopia, Tito works at the Tikur Anbesa Specialized Hospital in the capital, Addis Ababa, less than three kilometres away from a state-owned company that bottles liquid oxygen for industrial use.

“We are getting the oxygen from the largest oxygen plant in the country,” Tito says, “but we still face this problem.”

Tito’s case highlights one of the core challenges presented by oxygen cylinders: the difficulty of getting them from point A to point B and then back again for refill.

In Ethiopia, as in most countries, point A tends to be located in or around a big city, near the businesses that oxygen manufacturers serve – among them the iron and steel welders involved in construction.

Any obstacles occurring between points A and B – ranging from bad traffic to bad roads – tend to increase farther away from the city, as does the expense of transport and fuel.

“For hospitals that are farther away the situation is much worse,” Tito says.

Ethiopia is not the only country with this problem, numerous studies show that oxygen access is problematic world-wide. For example, a recent study published in the *Lancet* found that of facilities treating respiratory infections in sub-Saharan Africa, only around 1 in 5 had oxygen in Mauritania and 1 in 10 in Niger. Surveys carried out by the United States Agency for International Development paint a similar picture for countries in South America and South Asia.

A serious concern at any time, the global medical oxygen problem has been brought into sharper focus by the novel coronavirus disease (COVID-19) pandemic.

“It has been estimated that around one in five people with COVID-19 suffers respiratory distress sufficient to require oxygen therapy,” says Dr Priyanka Relan, a COVID-19 clinical management expert at WHO. “Without that therapy, COVID-19 can be fatal.”

In June, when the COVID-19 pandemic was expanding at a rate of around 1 million new confirmed cases a week, the World Health Organization (WHO) Director-General, Tedros Adhanom Ghebreyesus, stated that about 620 000 cubic metres of oxygen per day were required to meet demand. That corresponds to about 88 000 large oxygen cylinders.

“There’s (…) a real opportunity to close the oxygen access gap.”

Lisa Smith

One of the key questions governmental and non-governmental organizations are facing is how to get that oxygen to the people who need it. The methods attracting most interest involve generating oxygen at the point of use, thus avoiding problems with transport.

Pressure swing adsorption (PSA) oxygen plants are a good example. In the PSA process air is pushed through a vessel containing zeolite – a material that absorbs nitrogen – resulting in oxygen of sufficient purity for medical use. Relatively small, PSA plants can also take the form of containerized, skid-mounted units that can be delivered and installed onsite at hospitals.

A feature of paediatric pneumonia campaigns for several years, PSA plants are being considered with renewed interest in the context of COVID-19.

“We have been helping fund the installation and running of PSA plants for a number of years as part of efforts to provide healthcare facilities with reliable, affordable, locally-generated medical oxygen supplies,” says Jim Stunkel, vice president and director of programme operations at non-governmental organization Assist International.

Assist International has worked with local partners in Ethiopia, Kenya and Rwanda to install and run PSA plants. The Ethiopian plants were recently given extra support by Grand Challenges Canada (GCC) a Canadian nonprofit organization, which increased its investments in the plants to support Ethiopia’s COVID-19 response.

“The GCC investment enables the plants to run at 100% capacity, up from 70%, producing 120 cylinders a day, and servicing around 70 hospitals,” Stunkel says.

Though of proven value, PSAs require significant upfront and operational expenditure, and long-term maintenance, so may not be suitable for every setting.

“You need to service and maintain these units,” says Janet Diaz, head of the clinical unit in WHO’s emergency programme. “You don’t want to put a PSA in and then it stops working in five years because it wasn’t maintained properly.”

According to Diaz, WHO is focusing its efforts on oxygen concentrators, suitcase-sized devices that separate oxygen from ambient air on a much smaller scale.

“Concentrators are self-contained, relatively easy to install and maintain (although they do need maintenance) and have been shown to have a positive impact in Egypt, Malawi, and Papua New Guinea,” Diaz says.

WHO is currently negotiating with manufacturers around the world to buy oxygen concentrators for countries with acute needs. “To date, WHO has procured 14 000 concentrators, which have been sent to 120 low- and middle-income countries chosen because of their current outbreak status,” Diaz says, adding that the organization hopes to purchase a further 170 000 concentrators by the end of the year.

While concentrators are helping to meet increased demand during the pandemic, they also have limitations in the clinical setting.

The most commonly-used concentrators deliver a maximum of 10 liters of oxygen per minute, which may be enough to treat some severe COVID-19 patients, but is insufficient to treat the critically ill with the requisite ventilatory support.

Thus, as part of second phase response, WHO is now also supporting the scale-up of sustainable oxygen systems, with liquid oxygen and/or PSA systems in 10 countries.

“Concentrators (…) have been shown to have a positive impact in a number of low-income countries.”

Janet Diaz

Concentrators also require electricity, drawing about as much power as a small refrigerator. This is a problem in health facilities without access to power, of which there are many. The 2018 *Multi-tier framework survey* conducted by the Energy Sector Management Assistance Program, which collected data across 730 health facilities, including clinics and hospitals in Cambodia, Ethiopia, Myanmar, Nepal, Niger and Kenya, revealed that around 1 in 4 have no access to electricity.

Concentrators can be powered with standby generators, but these too can break down and require fuel to run. Innovators have been working on ways to operate concentrators off-grid, as part of efforts to improve access to oxygen therapy for paediatric pneumonia in remote locations.

Researchers from Global Health Uganda, a non-governmental organization, working with the University of Alberta in Canada, have developed a solar powered concentrator system called SPO_2_.

According to Dr Robert Opoka, a pediatrician and researcher at Makerere University in Kampala, the SPO_2_ system is being trialled at 20 facilities in Uganda, as well as in the Democratic Republic of the Congo.

“The SPO_2_ was developed to provide oxygen for children with severe pneumonia,” Opoka says, “but it is clear that the system could play an important role in the pandemic response if the government requires it.”

Not everyone thinks new approaches are the ultimate solution. “There’s also a real opportunity to close the oxygen access gap by expanding on market solutions that are already working,” says Lisa Smith, director of the COVID-19 respiratory care response coordination project at the Program for Appropriate Technology in Health (PATH).

One such solution is pooling the purchasing of oxygen. “Typically cylinders and concentrators are bought by individual hospitals, districts, counties, regions or even states,” Smith says. “If oxygen procurement were pooled, industrial providers would have more certainty about demand and charge less.”

Whichever approaches are chosen, they will have to be chosen and implemented swiftly.

“Meeting the demand for medical oxygen being driven by COVID-19 is going to require rapid review and swift, coordinated implementation of chosen solutions, reflecting local need,” says WHO’s Diaz. “Given the shortcoming of the oxygen delivery systems currently available, it is also going to require some new ideas.”

WHO has set up an expert panel of independent engineering and respiratory therapy experts and clinicians to assess new ideas and to determine which technologies WHO could procure in emergency situations, ahead of routine regulatory approval.

Finally, a comprehensive pandemic response is going to need more than just oxygen. It will require ensuring access to the devices needed to deliver oxygen to patients, ranging from pulse oximeters to ventilators, and health workers that are trained to use such devices effectively.

However daunting, the investment of time and resources required to meet the demands imposed by COVID-19 does offer a silver lining: as the pandemic subsides, care for people with severe pneumonia and other conditions needing oxygen therapy, will improve.

**Figure:**
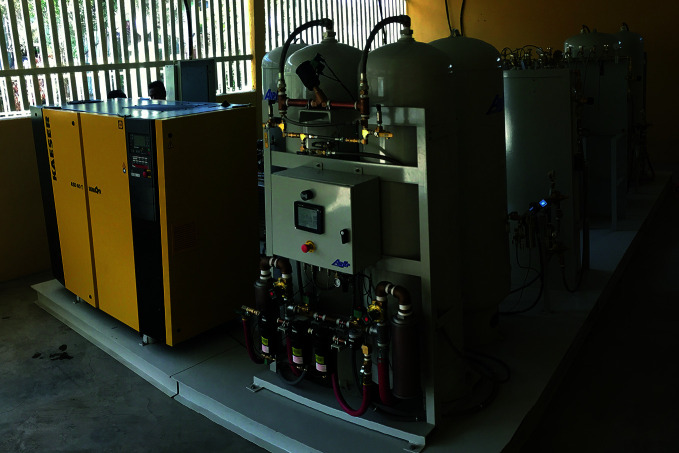
One of two new pressure swing adsorption plants providing medical oxygen to hospitals in the Amhara Region of Ethiopia.

Courtesy of Assist International

**Figure:**
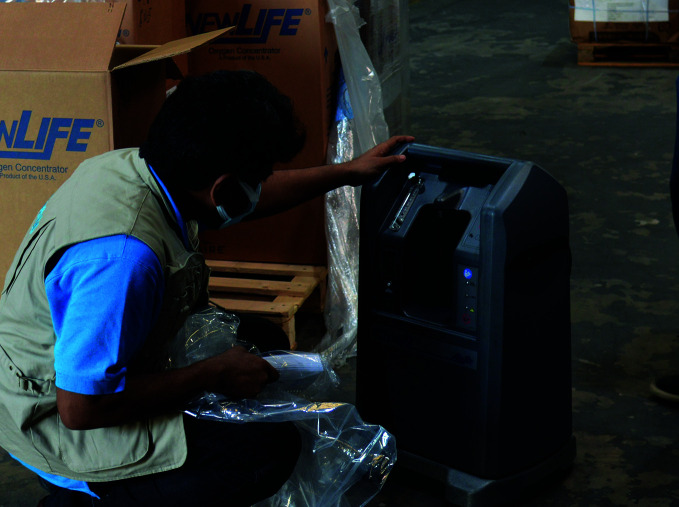
A UNICEF staff member inspects oxygen concentrators destined for use in health facilities in Ghana.

E. Offeibea Baddo/UNICEF

